# Pre-translational regulation of luteinizing hormone receptor in follicular somatic cells of cattle

**DOI:** 10.1016/j.anireprosci.2015.09.019

**Published:** 2015-12

**Authors:** P. Marsters, N.R. Kendall, B.K. Campbell

**Affiliations:** a University of Nottingham, Division of Child Health, Obstetrics and Gynaecology, Queen's Medical Centre, Nottingham NG7 2UH, United Kingdom; b School of Veterinary Medicine and Science, University of Nottingham, Sutton Bonington Campus, Sutton Bonington, Leicestershire LE12 5RD, United Kingdom

**Keywords:** Luteinizing hormone receptor, Pre-translational regulation, Follicle, Granulosa cells, Theca cells, Variable deletion site

## Abstract

Differential regulation of LHR in theca cells (TC) and granulosa cells (GC) is important for normal follicular development. Unlike TC, GC only acquire LH-responsiveness during the later stages of antral follicle development. This study tested the hypothesis that differential LH-responsiveness in these two cell types may be due, in part, to shifts in cellular patterns of alternatively spliced *LHR* mRNA transcripts which may not be obvious from analysis of total *LHR* gene expression. It also further explored the role of translation inhibition by an *LHR* binding protein (LHBP), normally associated with the production of endogenous cholesterol. *LHR* mRNA variation arises as a result of the alternative splicing of two variable deletion sites (VDS) designated 5′ VDS and 3′ VDS, and it was proposed that differences in cell sensitivity to LH may be due in part to variations in the pattern of the mRNA expression of the receptor variants. The outcomes of the present study support a dynamic multi-facetted regulation of *LHR* during pre-translation. Not only did the ratio between variants change during antral follicle growth and *in vitro* cell differentiation but also between TC and GC. Regulation could also be linked to LH concentration feedback mechanisms as the absence of LH caused cultured TC to markedly up-regulate amounts of *LHR* mRNA. In both TC and GC, LHR mRNA was greatly reduced after treatment to block mevalonate production in the *de novo* cholesterol pathway, adding further support for a regulatory mechanism linked to enriched cellular amounts of mevalonate kinase.

## 1 Introduction

The pituitary gonadotrophins, luteinising hormone (LH) and follicle stimulating hormone (FSH) signal *via* specific membrane bound receptors. FSH receptors occur exclusively on the GC of ovarian follicles from primary through to the pre-ovulatory stages of follicle development (McNatty et al., 1999), whereas, LH receptors (LHR) first develop in the cells of the theca interna at the tertiary stage of development and this pattern of gene expression is maintained through to the pre-ovulatory stage (Richards, 1994). In addition, it is well established that the GC of large oestrogenic antral follicles also develop LHR (Hampton et al., 2004, Campbell et al., 2003, Webb et al., 1999) and this suggests that GC responsiveness to LH may be important in the later stages of follicle growth in mono-ovulating species. It has been suggested that the maintenance of follicular dominance may rely on a follicle's heightened LH- and reduced FSH-responsiveness (Campbell and Baird, 2001). LH receptors also play a critical role in supporting progesterone secretion by the corpus luteum (Niswender, 2002). Overall, the responsiveness of follicular somatic cells to LH has attracted a great deal of research interest. However, the mechanisms underlying regulation of its cellular responses still remain relatively poorly understood.

Classic LH-binding studies (Carson et al., 1979, Webb and England, 1982, Peng et al., 1991) and *in situ* hybridisation studies (Xu et al., 1995), demonstrated that only GC from large pre-ovulatory follicles had detectable LH-binding capacity. In contrast *LHR* mRNA has been detected in both GC and TC of antral phase follicles in the dissectible (by eye) range (greater than around 1.0 mm) in sheep (Abdennebi et al., 2002) and cattle (Robert et al., 2003). However the outcomes of mRNA studies utilising quantitative approaches are more consistent with the earlier protein reports in that *LHR* mRNA was found to be minimal in GC from large follicles (≥8 mm follicles, Bao et al., 1997, Evans and Fortune, 1997, Nogueira et al., 2007).

**Fig. 1:**
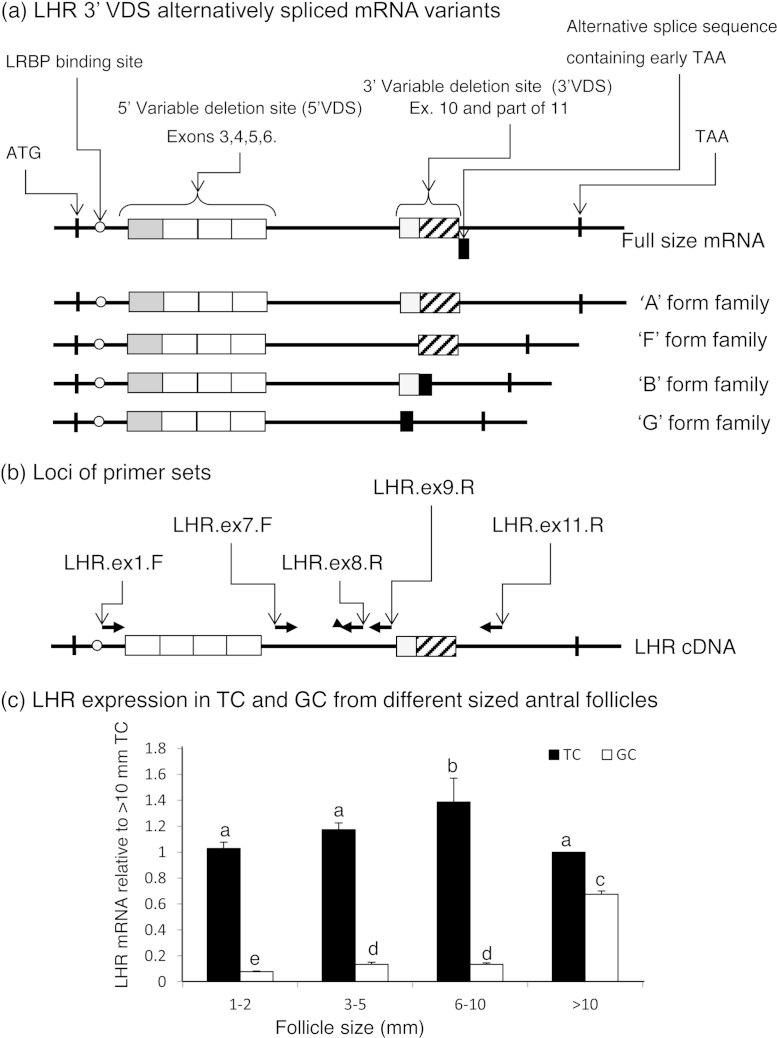
Schematic representation of the *LHR* full size mRNA (a) showing the positions of the 5′ and 3′ variable deletion sites (VDSs) and other important loci. Alternative splicing of the 3′ VDS determines the variant family (also shown), members of which may undergo further splicing combinations within the 5′ VDS. Also shown is a schematic representation of the *LHR* cDNA (b). Directional arrows depict the PCR primer target sites in relation to the two variable deletion sites (5′ VDS and 3′ VDS, respectively, F denotes forward primer and R denotes reverse primer and ‘ex’ denotes exon). Total *LHR* mRNA is compared in TC and GC (c) of cattle utilising *LHR*.ex7.F/*LHR*.ex9.R universal primer set. The graph shows the mean outcomes ± SEM of at least three experiments performed in duplicate (differences are denoted by different lower case letters).

The propensity of *LHR* to generate a number of alternatively spliced mRNA transcripts has to date not been shown to equate to the production of an extended and functionally diverse family of LHR variants. However it is hypothesised that the differential expression of LHR mRNA in GC and TC could be a pre-translational mechanism for regulating LH-responsiveness. In humans eight *LHR* mRNA isoforms have been catalogued (Reinholz et al., 2000). Alternatively spliced transcripts vary due to deletion of all or part of two variable coding regions: one incorporating Exon 3 (in humans the last 25 bases of Exon 2 are included) to Exon 6, and the other Exon 9 to the first 266 bases of Exon 11 (Fig. 1). To avoid confusion, in the present study these were designated as 5′ and 3′ variable deletion sites (VDS), respectively. Several studies concentrating on the 3′ VDS have described four mRNA isoforms, which are present in the ovary of large domestic ruminants and several other species. These have been designated ‘A’, ‘B’, ‘F’, and ‘G’ isoforms in sheep (Bacich et al., 1999; Fig. 1) but appear to have corresponding isoforms in a number of other species including cattle, pigs, rats and humans (cattle, Kawate and Okuda, 1998; pigs, Loosfelt et al., 1989; rats, Bernard et al., 1990; humans, Reinholz et al*.*, 2000). However, these variant designations, are based only on analysis of the 3′ VDS region of the *LHR* gene sequence and do not comment on the presence or absence of upstream regions. Thus the so called ‘A’, ‘F’, ‘B’, and ‘G’ 3′ VDS variants may be more accurately described as members of alternative splicing families which may have also undergone variation within the 5′ VDS (Fig. 1). Moreover *LHR* mRNA which has the whole of Exon 3 deleted has been identified in cattle follicles (Nogueira et al., 2007).

Full LHR functionality, which includes ligand specificity and nuclear signalling capacity, appears to be conferred only by translation of the undeleted mRNA splice variant which would incorporate the ‘A’ form 3′ VDS (Ji and Ji, 1991). No role has been suggested for either the soluble ‘B’ form (Bacich et al., 1999) or for any of the other putative products of the variously deleted *LHR* mRNA isoforms. However, this apparent lack of functionality may not exclude the possibility that variant mRNA transcripts have a role in regulating cellular sensitivity to LH and this, therefore, remains an important research goal. A candidate mechanism for regulation has been described in rat and primate ovaries. Mevastatin kinase (MVK), an important enzyme in the *de novo* cholesterol pathway, binds to *LHR* mRNA and inhibits translation (Menon et al., 2009, Nair and Menon, 2005, Wang et al., 2007). It is, therefore, hypothesised that differential regulation of functionally intact LHR in TC and GC may involve multiple layered mechanisms during pre-translation, which incorporate alternate splicing and translation blocking by MVK.

The central aim of the present study was to determine whether sensitivity to LH, in follicular somatic cells of cattle harvested at different stages of antrum development and during *in vitro* cellular differentiation, involves fluctuations in the pattern of relative amounts of *LHR* splice variant mRNA and to investigate the case for a multi-layered regulatory system which includes *LHR* translation inhibition.

## 2 Materials and methods

Unless otherwise indicated all reagents were purchased from Sigma–Aldrich Co. Ltd., Poole, Dorset, UK. Mevastatin was made up to a 0.5 mM (100×) stock solution in 1 N NaOH by incubating at 50 °C for 1 h. A working concentration of 5 μM was determined as being in the optimal range from previous studies (Kwintkiewicz et al., 2006).

### 2.1 Tissue collection and TC and GC isolation

Cattle ovaries were taken from freshly slaughtered abattoir animals (>30 months old) and maintained at 37 °C in a collection/dissecting buffer of Dulbecco's modified eagle medium (DMEM) with 1% penicillin (5000 U/ml)/streptomycin (5 mg/ml) solution, 1% Fungizone^®^ (amphotericin B – 250 μg/ml), and 20 mM HEPES. Follicles (greater than 1 mm in diameter) were blunt dissected cleanly to avoid extraneous tissue carryover. Follicles, which appeared to be morphologically normal were then separated into size groups (1–2 mm, 3–5 mm, 6–10 mm and >10 mm). After hemisection of the follicles, in each size group GC and TC were separated by gentle flushing and scraping of the follicle walls in calcium and magnesium free Dulbecco's phosphate saline buffer (DPBS). The pooled GC and TC were then washed twice in DPBS by centrifuging at 800 *g* for 10 min and re-suspended in RNeasy™ RTL lysis buffer (QIAGEN) containing 1% β-mercaptoethanol.

### 2.2 GC culture

Isolated GC from the less than 5 mm subsets of small antral follicles (pre-steroidogenic or minimally steroidogenic) were also selected for culture, and in accordance to a specialised protocol (Gutierrez et al., 1997) the cells were washed twice in Dulbecco's phosphate saline buffer (DPBS) by centrifuging at 800 *g* for 10 min and then re-suspended in a culture medium of McCoys 5a with sodium bicarbonate containing 250 μg/ml bovine serum albumin (BSA), 1% penicillin (5000 U/ml)/streptomycin (5 mg/ml) solution, 15 mM l-Glutamine, 20 mM HEPES, 10 μg/ml Transferrin (Merck Biosciences Ltd.), 4 ng/ml Selenium (sodium selenite), 100 ng/ml testosterone, 1 ng/ml insulin-like growth factor type 1 (IGF-1) LR3, 10 ng/ml Insulin, and 1 ng/ml ovine FSH (NIAMDD-FSH-S20) (Gutierrez et al., 1997). The viability of the cells was determined by trypan blue exclusion and cells were seeded in 24-well Nunclon™ microtitre plates (Nalge Nunc International) at the rate of 5 × 10^5^ viable cells per well in 1 ml of culture medium. A sample of cells was retained as the zero hrs time point sample, and stored at −20 °C, in 150 μl of RNeasy™ RTL buffer (Qiagen) containing 1% β-mercaptoethanol.

### 2.3 TC culture

Isolated TC from the same 3–5 mm subset of small antral follicles were subjected to enzymatic dispersion as previously described (Campbell et al., 1998) by incubating for 45 min in 10 ml of calcium and magnesium free DPBS containing 50 mg of collagenase, 10 mg of hyaluronidase, 10 mg of protease and 100 μl of foetal calf serum. Following dispersion the pooled TC were washed twice by centrifuging at 800 *g* for 10 min and then re-suspended in DMEM:Hams F12 culture medium of containing sodium bicarbonate and HEPES, 1 mg/ml BSA, 1% penicillin (5000 U/ml)/streptomycin (5 mg/ml) solution, 5 mM l-Glutamine, 10 μg/ml Transferrin, 4 ng/ml Selenium (sodium selenite), 10 ng/ml IGF-1 LR3, 10 ng/ml Insulin, and 0.1 ng/ml ovine LH (NIAMDD-LH-S26; Campbell et al., 1998). Viability of the cells was determined by trypan blue exclusion and they were seeded in 24-well Nunclon™ microtitre plates at the rate of 5 × 10^5^ viable cells per well in 1 ml of culture medium. A sample of cells was retained as the zero hrs time point sample, and stored at −20 °C, in 150 μl of RNeasy™ RTL buffer containing 1% β-mercaptoethanol.

### 2.4 GC and TC time courses

The GC and TC were then maintained in a humidified atmosphere of 95% air and 5% carbon dioxide, at 37 °C, over a series of time points (16, 24, 48, 96, 144, 192 h). Groups of replicates were set up where cells were either left untreated or were treated with mevastatin (final concentration of 5.0 μM), to block the *de novo* cholesterol synthesis pathway, or were left untreated. A working concentration of 5.0 μM was selected from the previously determined optimal range 3.0–10.0 μM (Izquierdo et al., 2004, Kwintkiewicz et al., 2006). The cells underwent media changes every 48 h but to avoid disturbing the cells only 80% of the media volumes were changed. At each time point the media was removed from the appropriate wells and stored at −20 °C for later analysis by RIA, and the cells were washed in sterile DPBS and taken off in 350 μl/well of RNeasy™ RTL buffer (Qiagen) containing 1% β-mercaptoethanol. The pooled lysates from each time point were stored at −20 °C for later analysis by semi-quantitative RT-PCR.

### 2.5 Semi-quantitative RT-PCR

Total RNA was isolated from the time course samples using the RNeasy™ Mini Kit (QIAGEN) and protocol. First strand cDNA synthesis was performed using Revertaid™ H-minus reverse transcriptase (MBI). Random hexamers (Promega) were used to prime the cDNA strand as an 18S internal standard (QuantumRNA™ 18S rRNA Universal Internal Standard; Ambion) was used. To detect the different transcript variants, conventional, endpoint PCR amplification was performed using cDNA-distinguishing primers (see next section). The PCR products were electrophoresed on 2% agarose gels containing ethidium bromide, and visualised under UV light. Subsequent analysis was by laser densitometry. The PCR products from parallel gels were excised and purified using QIAquick™ Gel Extraction Kit (QIAGEN). Product identities generated by each primer set were subsequently confirmed by sequencing.

### 2.6 Rationale for use of conventional PCR and primer selection

Primers were designed from bovine *LHR* sequence (GenBank, accession No. U20504) and were selected in all cases to amplify regions of *LHR* cDNA, which contained at least one exon/exon boundary to ensure recognition of contaminating genomic (intron containing) DNA. Initially a set of primers was designed to amplify the region between Exon 1 and Exon 11 (*i.e.* including both variable deletion sites; Fig. 1). However given that the two deletion sites involve six potential regions for deletion between them the permutations of possible variants are numerous. Even though a relatively smaller number of splice variants appear to be transcribed in the follicular somatic cells of cattle, these nevertheless generate too many PCR products for meaningful analysis. Because Northern blot probes can also elucidate targets on multiple LHR splice variant transcripts, Northern analysis was also ruled out as a viable alternative method. Therefore, it was decided to conduct separate surveys for each of the VDSs by RT-PCR. Primers were utilised (Table 1 and Fig. 1) which amplified the 5′ VDS (*LHR*.ex1.F/*LHR*.ex8.R) and the 3′ VDS (*LHR*.ex7.F/*LHR*.ex11.R) regions and a region common to all splice variants (*LHR*.ex7.F/*LHR*.ex9.R).

### 2.7 Radioimmunoassays

Oestradiol-17β (E2; (Marsters et al*.*, 2003) and androstenedione (A4; (Campbell et al., 1998) concentrations in culture media from each time-point were assayed by double antibody radio-immuno assay (RIA), as previously described. The sensitivities of the E2 and A4 assays were both 39 pg/ml (∼90% of zero binding value) and the inter- and intra-assay coefficients of variation were <14% and <10%, respectively in both cases.

### 2.8 Statistical analysis

Steroid concentrations at the 96, 144, 192 h time points were corrected to exclude carry over from previous time points after 80% media change every 48 h. This assumed that the concentration of the 20% carry over volume from the previous time-point did not alter greatly over 48 h. To ensure that the PCR products from each sample were comparable the PCRs were halted during the exponential amplification phase and product signal intensities were arbitrarily quantified using densitometry and normalised against the *18S* endogenous control. When comparing *LHR* mRNA to steroid production in the time courses, amounts at each time point were calculated relative to amounts at time zero. The results shown are the means ± SEM of at least three independent experiments performed in duplicate. ‘Repeated measures and 1-way ANOVA’ were performed using SPSS (version 16.0) software to determine the level of significance of the effects of treatment and time of culture amount and hormone production, with ‘function’ used to compare data to the previous time point.

## 3 Results

### 3.1 Total LHR mRNA in TC and GC taken from various size cattle follicles

Using a PCR primer set which targeted all *LHR* splice variants (*LHR*.ex7.F/*LHR*.ex9.R; Table 1 and Fig. 1a and b) to determine the relative amounts of *LHR* mRNA in TC and GC taken from various sized follicles it was found (Fig. 1c) that there were marked differences between the two cell types, with relative amounts of *LHR* mRNA in TC from follicles in the 1–2 mm, 3–5 mm, 6–10 mm and >10 mm pools being greater than in GC from the same sized follicles. The relative amounts of *LHR* mRNA in TC from 6 to 10 mm follicles were markedly greater (*P* < 0.001) than in the other groups. In contrast, the amount of *LHR* mRNA was barely measurable in GC from 1 to 2 mm follicles but notably (*P* < 0.01) greater in the 3–5 mm and 6–10 mm diameter follicle groups and markedly (*P* < 0.001) greater in the >10 mm in diameter follicles.

### 3.2 Total LHR mRNA in cultured TC compared to production of A4

**Fig. 2:**
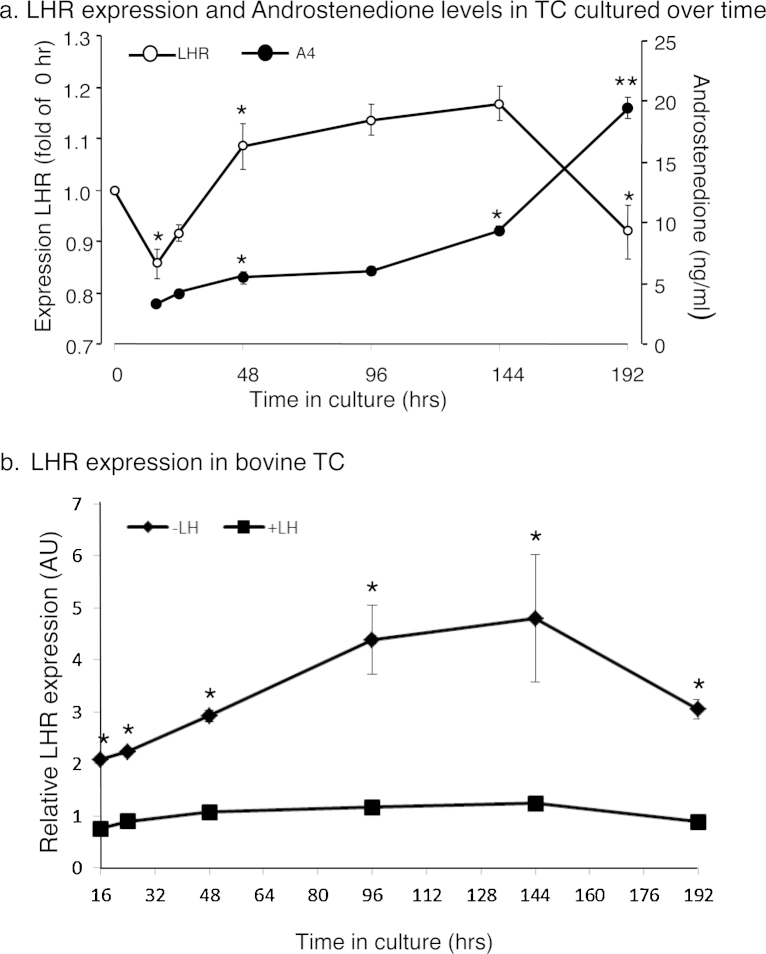
Total LHR variant mRNA in TC of cattle, recovered from 3 to 5 mm follicles and cultured over time under LH stimulation, compared to production of A4 in the same cells (^a^asterisks denote a difference compared to the preceding time point, *denotes *P* < 0.05 and **denotes *P* < 0.01). The total relative amounts of *LHR* variant with stimulation by LH is also shown compared to without LH in TC recovered from 3 to 5 mm follicles and cultured over time (^b^asterisks denote a difference between treatment groups at the same time point). The graphs depict the means ± SEM of at least three separate experiments performed in duplicate.

Using the same PCR primer set which targeted all LHR splice variants (*LHR*.ex7.F/*LHR*.ex9.R; Table 1 and Fig. 1a and b) to obtain a profile for relative amounts of *LHR* mRNA in cultured TC over time it was found that amounts in the presence of 1 ng/mL of LH were markedly decreased (*P* < 0.05) immediately after seeding (Fig. 2a). However after 16 h in culture, relative amounts markedly increased (*P* < 0.05) to the 48 h time point after which time a trend of continued increase was not statistically significant and after 144 h of culture relative amounts markedly decreased (*P* < 0.05) over the final 48 h of the culture period. Amounts of A4 in the corresponding TC culture media increased steadily over time (*P* < 0.05) with a marked increase from 144 to 192 h (*P* < 0.001). The marked increase in A4 production coincided with the marked decrease in *LHR* mRNA. Removal of LH from the TC culture medium resulted in marked increases in *LHR* mRNA at all of the time points with amounts peaking at around 96 h of culture to almost a four-fold increase (*P* < 0.01) compared with amounts in the presence of LH (1 ng/mL, Fig. 2b).

### 3.3 Total LHR mRNA in cultured GC compared to production of E2

**Fig. 3:**
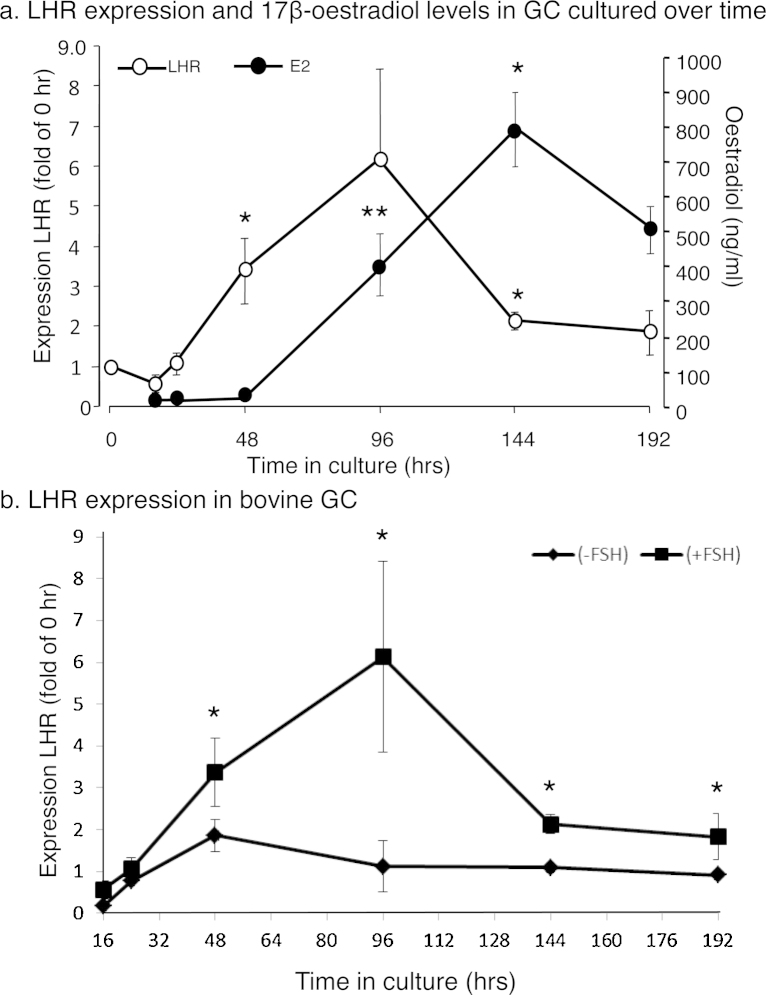
Total *LHR* variant in GC of cattle, recovered from 3 to 5 mm follicles and cultured over time under FSH and testosterone stimulation, compared to production of E2; in the same cells (^a^asterisks denote a difference compared to the preceding time point, *denotes *P* < 0.05 and **denotes *P* < 0.01). The total amounts of *LHR* variant under stimulation by FSH and testosterone is also shown compared to without FSH in GC recovered from 3 to 5 mm follicles and cultured over time (^b^asterisks denote a difference between treatment groups at the same time point). The graphs depict the means ± SEM of at least three separate experiments performed in duplicate.

When the same PCR primer set (*LHR*.ex7.F/*LHR*.ex9.R) was used to analyse total relative amounts of *LHR* mRNA in GC cultured over time, in the presence of 1 ng/mL of FSH, there was no significant variation over the first 24 h in culture (Fig. 3a). However, over the next 24 h relative amounts of LHR markedly increased (*P* < 0.05), and though not significantly this trend continued beyond the 48 h time point, peaking after 96 h of culture, at about six-fold greater than the initial amounts. Beyond this point total relative amounts of *LHR* mRNA markedly decreased (*P* < 0.05) over the next 48 h to about two-fold of starting amounts, and remained unchanged thereafter. Corresponding production of E2 by the cultured GC was minimal over the first 48 h (Fig. 3a), but thereafter increased markedly (*P* < 0.01) over the next 48 h, peaking at 144 h of culture to about eight-fold the amounts at the start of the culture period (*P* < 0.05), before decreasing to the 192 h time point. Also, in contrast to removal of LH in TC cultures, the removal of FSH from the GC culture medium (Fig. 3b) resulted in a marked decrease in *LHR* mRNA (*P* < 0.05) at all time-points except 16 and 24 h of culture.

### 3.4 LHR 5′ VDS in TC and GC taken from various sized follicles

**Fig. 4:**
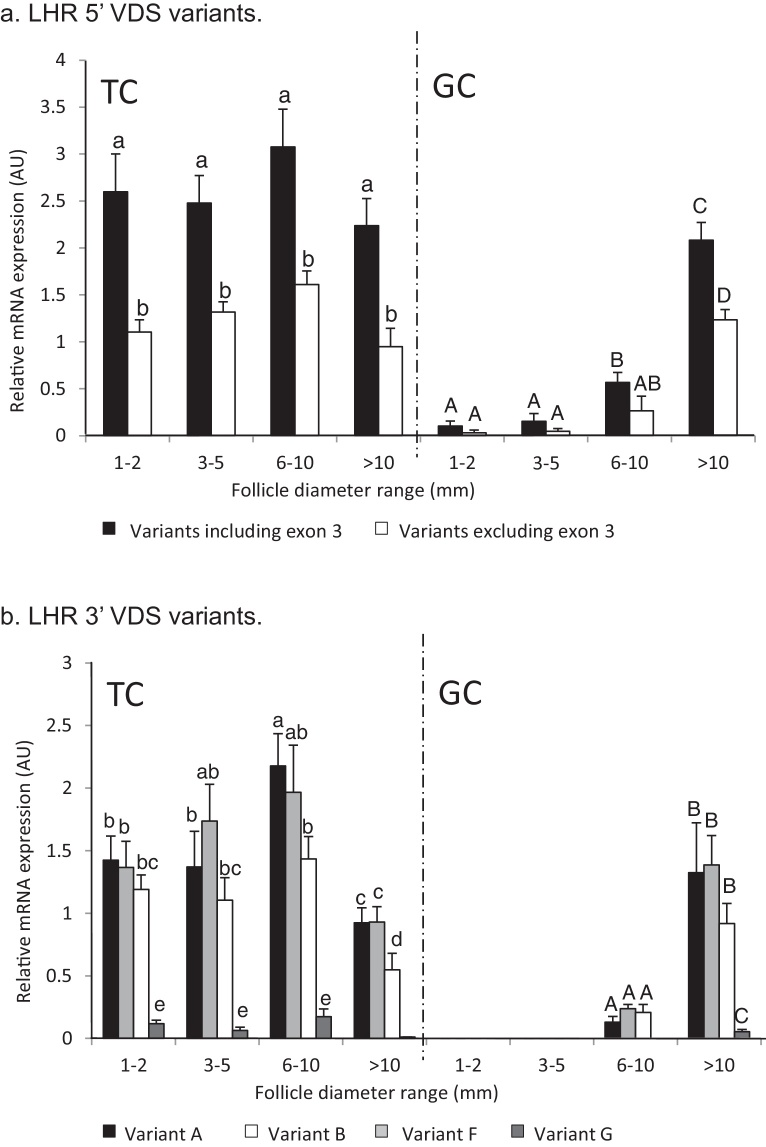
Relative amounts of *LHR* splice variants (^a^5′ variants; ^b^3′ variants) in TC and GC of cattle taken from various size antral follicles (no common letters denote differences, *P* < 0.05). The graphs depict the means ± SEM of at least five separate follicle collections and each pool contained at least 12 follicles.

PCR amplification of the 5′ VDS region using the *LHR*.ex1.F/*LHR*.ex8.R primer set (Table 1 and Fig. 1) detected two possible 5′ VDS families of *LHR* mRNA splice variants in TC and GC from all antral follicle size groups (Fig. 4a). These were variants incorporating an intact 5′ VDS, and a possible 424 bp truncated 5′ VDS family of splice variants was also noted. Subsequent sequencing revealed that these shortened splice variants had undergone deletion of the entire Exon 3. Though relative amounts of both *LHR* mRNA variants remained unchanged in TC irrespective of follicle size, the amounts of the intact 5′ VDS variants in all size groups were consistently greater (*P* < 0.01) than the truncated forms. In contrast, only GC from follicles ≥10 mm in diameter (*P* < 0.01) contained the intact variants in greater amounts than the forms with Exon 3 missing. However, the amounts of both of the 5′ VDS splice alternatives were markedly greater in GC from the ≥10 mm follicles than in all other size groups (*P* < 0.01).

### 3.5 LHR 3′ VDS in TC and GC taken from various sized follicles

A number of LHR mRNA splice variations were detectable using the primer set *LHR*.ex7.F/*LHR*.ex11.R (Fig. 1b), which amplifies the region containing the 3′ VDS. After sequencing, these were confirmed as the A, F, B, and G, 3′ VDS variant forms (Fig. 4b). The full length *LHR* mRNA containing these could potentially either incorporate an intact 5′ VDS or have Exon 3 excluded. The ‘A’ isoform variants incorporate an intact 3′ VDS whereas the others have undergone some sequence deletions (Fig. 1a and b) at this site. In TC from all follicle sizes less than 6 mm, there was no difference in relative amount LHR mRNA between the ‘A’, ‘B’ and ‘F’ isoforms, although in the 6–10 mm and the >10 mm follicle groups relative amounts of variant ‘A’ were greater (*P* < 0.01 and 0.001, respectively) than the ‘B’ variant. All three splice variant forms were in lesser in amounts (*P* < 0.01) in TC from >10 mm follicles than in the other follicle size groups. Variants with the ‘G’ form deletions were minimal in amounts (*P* < 0.001) in the TC of all follicle size groups and were virtually undetectable in TC from <10 mm follicles. In contrast, relative amounts of all of the *LHR* variants in GC taken from follicles <6 mm, was below the threshold for detection or accurate quantification. In GC from 6 to 10 mm follicles, relative amounts of *LHR* mRNA splice alternatives ‘A’, ‘B’ and ‘F’ were in the detection range but differences in amounts between variants were not significant. Relative amounts of all three were markedly less (*P* < 0.01) than in GC taken from >10 mm follicles. Furthermore in cells taken from the >10 mm follicles the relative amounts of all variants appeared greater in GC than in TC although differences were not statistically significant. Variant G isoforms were only detectable in the GC from this size group of follicles in minimal amounts.

### 3.6 LHR 5′ VDS in TC cultured over time

**Fig. 5:**
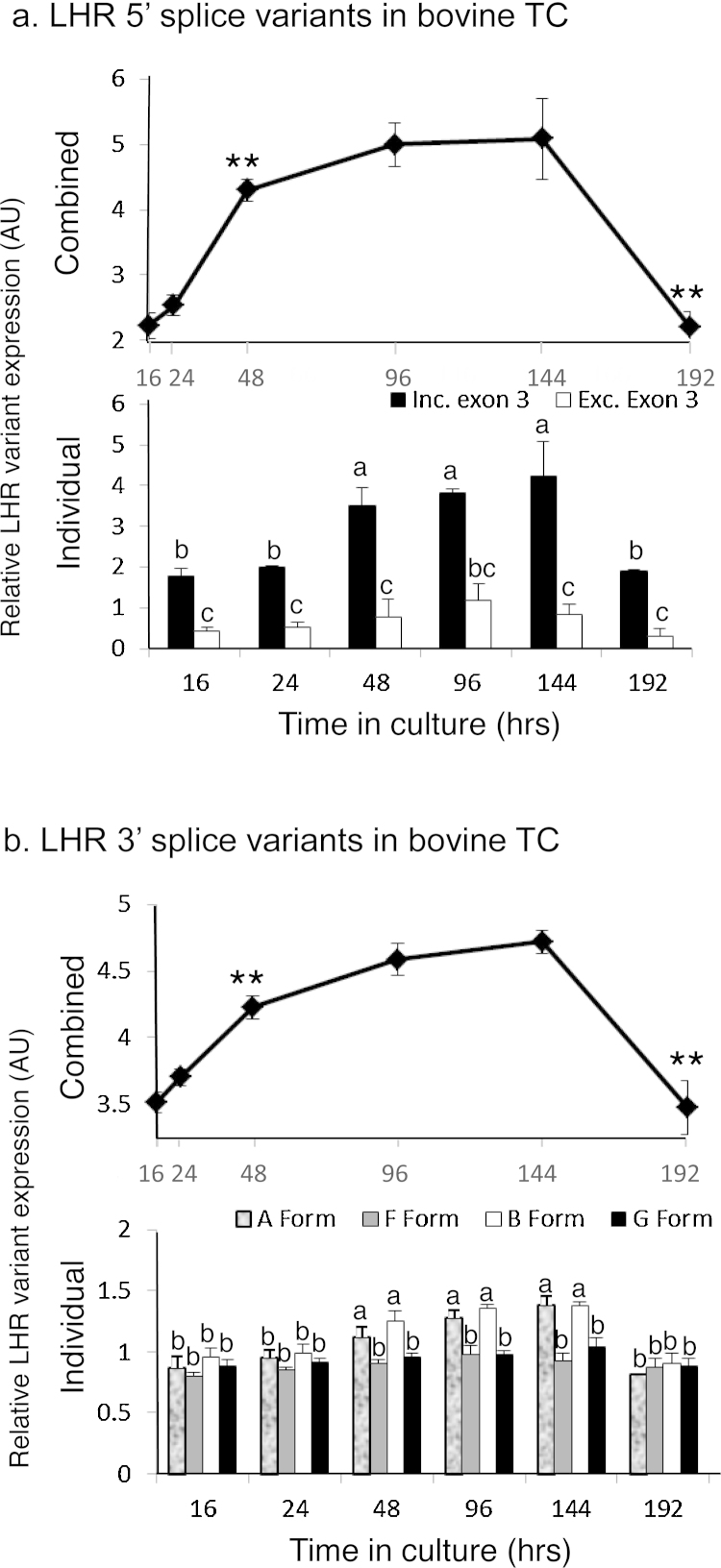
Relative amounts of *LHR* splice variants (^a^5′ variants; ^b^3′ variants) in TC of cattle, recovered from 3 to 5 mm follicles and cultured over time (no common letters denote differences, *P* < 0.05). In both cases the upper graphs show the combined amounts of variant (asterisks denote difference compared to the preceding time point, *denotes *P* < 0.05 and **denotes *P* < 0.01). The graphs depict the means ± SEM of at least three separate experiments performed in duplicate.

When the primer pair *LHR*.ex1.F/*LHR*.ex8.R was used, in TC of cattle cultured over time, to amplify *LHR* mRNA isoforms having the 5′ VDS (Fig. 1b), the same two 5′ VDS splice variations were detected as in cells taken from various sized antral follicles. Relative amounts of total *LHR* mRNA (Fig. 5a, upper graph) had a time-course profile of an increase in amounts after 16 h of culture, with the amount at the 48 h time point being greater (*P* < 0.01) than at the previous time point. This trend continued for 96 h reaching a peak at 144 h of culture. Beyond this time point amounts markedly decreased to 192 h of culture (*P* < 0.01). However there was a marked difference in amounts of the two variant mRNA forms at all of time points (Fig. 5a, lower graph) with the Exon 3-deleted variants being less (*P* < 0.05) than the intact isoform *LHR* mRNA. However, relative amounts of the intact form of mRNA peaked at 144 h of culture, 48 h after the truncated form.

### 3.7 LHR 3′ VDS in TC cultured over time

Utilising the primer set *LHR*.ex7.F/*LHR*.ex11.R, which straddle the 3′ VDS (Fig. 1b), it was again demonstrated that ‘A’, ‘B’, ‘F’, and ‘G’ splice variant family variants were present in TC cultured over time (Fig. 5b). The relative amounts of total *LHR* mRNA (Fig. 5b, upper graph) again followed the same trend as that determined for *LHR*.ex1.F/*LHR*.ex8.R and the *LHR*.ex7.F/*LHR*.ex9.R primer sets, where marked increases were observed after 16 h of culture until the 48 h time point which was greater (*P* < 0.01) than at the preceding time point. The peak amount occurred at 144 h of culture and there was a marked decrease over the next 48 h (*P* < 0.01). When the relative amounts of the 4 variant mRNAs were compared (Fig. 5b, lower graph) these were in a similar amount to 24 h of culture. However between 48 and 144 h of culture the amounts of ‘A’ and ‘B’ forms markedly increased (*P* < 0.05) compared to the other variants which remained in similar amounts as those at the earlier time points. Amounts of all variants after 192 h of culture returned to amounts at the 16 and 24 h time points (*P* < 0.01 for variant ‘A’ and ‘B’).

### 3.8 LHR 5′ VDS in GC cultured over time

**Fig. 6:**
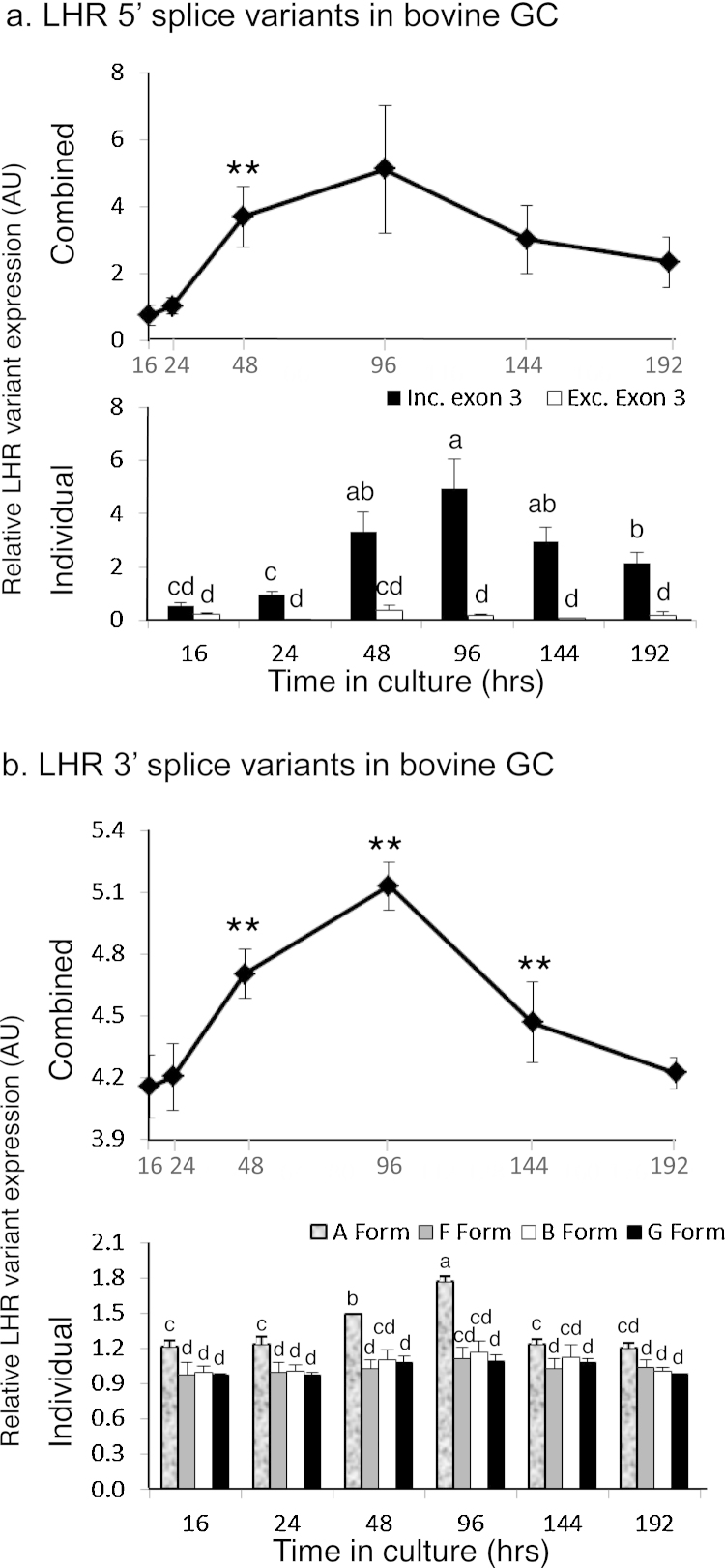
Relative amounts of *LHR* splice variants (^a^5′ variants; ^b^3′ variants) in GC of cattle, recovered from 3 to 5 mm follicles and cultured over time (no common letters denote differences, *P* < 0.05). In both cases upper graphs show the combined amounts of variant (asterisks denote difference compared to the preceding time point, *denotes *P* < 0.05 and **denotes *P* < 0.01). The graphs depict the means ± SEM of at least three separate experiments performed in duplicate.

The primer pair *LHR*.ex1.F/*LHR*.ex8.R was also utilised, in GC of cattle cultured over time, to amplify *LHR* mRNA isoforms with the 5′ VDS (Fig. 1). Again the same two 5′ VDS splice variations were detected as in TC over time and in cells taken from various sized antral follicles. Relative amounts of total *LHR* mRNA (Fig. 6a, upper graph) were again similar to the previously reported time-course profiles. There was a trend of increase after 16 h of culture such that at the 48 h time point there had been a marked increase (*P* < 0.01) compared with the preceding time point. However unlike the case in TC, amounts of *LHR* mRNA in GC peaked after 96 h of culture and then decreased thereafter. As with TC the amount of the individual variant mRNA isoforms was markedly different at all time-points other than at 16 h (Fig. 6a, lower graph) with the Exon 3-deleted variants being in lesser amounts (*P* < 0.01) than the intact isoform LHR mRNA. After 24 h of culture, the relative amount of the intact form increased disproportionately to the truncated form with the greatest amount being detected at the 96 h time point.

### 3.9 LHR 3′ VDS in GC cultured over time

The primer pair *LHR*.ex7.F/*LHR*.ex11.R was also utilised, in GC of cattle cultured over time, to amplify *LHR* mRNA isoforms with the 3′ VDS (Fig. 1). Again, the four 3′ VDS splice variants, ‘A’, ‘B’, ‘F’ and ‘G’, were detected as in TC over time and in cells taken from various sized antral follicles. Relative amounts of total *LHR* mRNA (Fig. 6b, upper graph) were similar to the previously reported time-course profiles. There was a trend for an increase after 16 h of culture which peaked at 96 h with the increases at both the 48 and 96 h time points being greater than at the preceding time points (*P* < 0.01). After this time point, relative amounts of *LHR* decreased markedly (*P* < 0.01) to 144 h of culture and then less so until the end of the time course. Comparing the relative amounts of the individual splice variants at each time point isoforms ‘B’, ‘F’ and ‘G’ were consistently similar while up until 96 h of culture Variant ‘A’ was consistently greater (*P* < 0.05) than the other mRNA isoforms (Fig. 6b, lower graph). The differences were greatest at the 48 (*P* < 0.01) and 96 h (*P* < 0.001) time points.

### 3.10 Effect of mevastatin on LHR mRNA

**Fig. 7:**
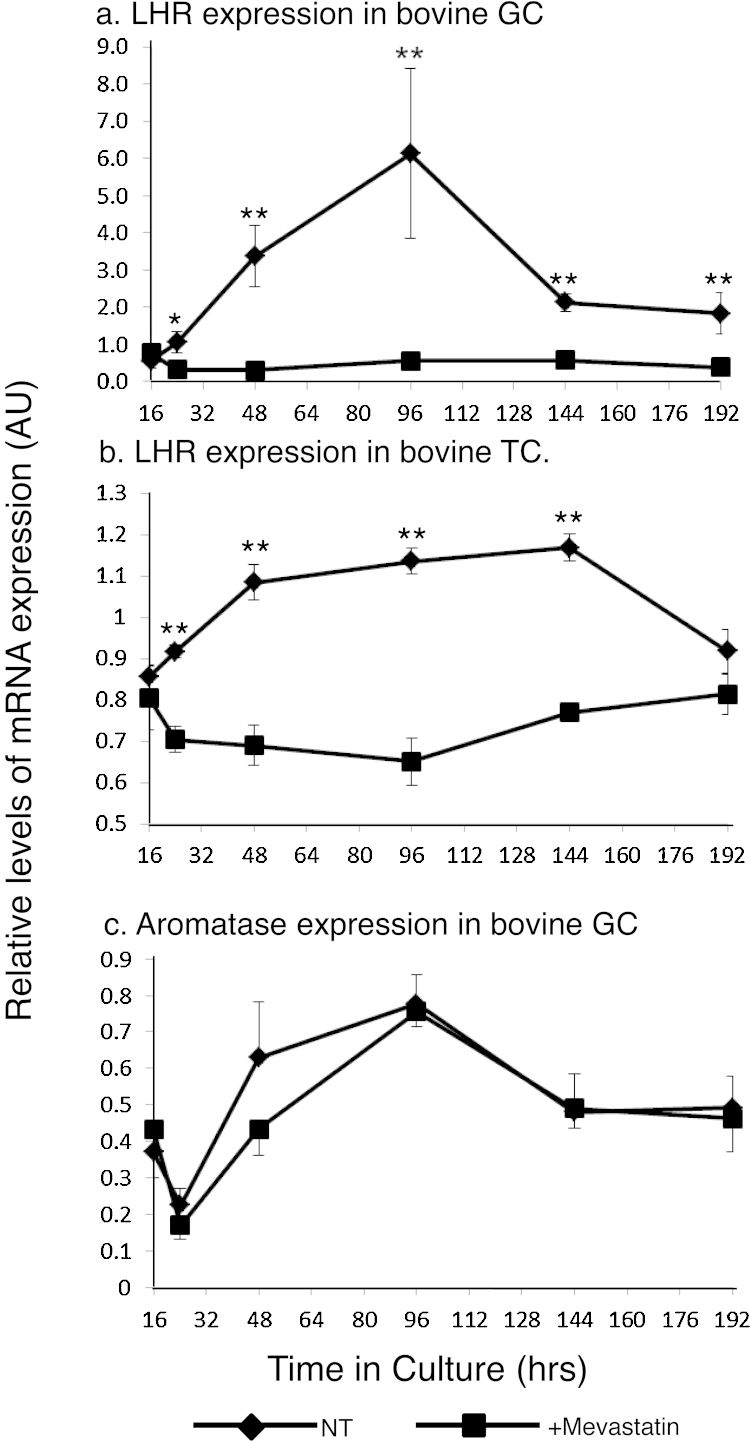
Total relative amounts *LHR* variant in follicular somatic cells of cattle cultured over time either in the presence of or without mevastatin treatment (^a^GC; ^b^TC). Also shown is the amount of *CYP19* in GC cultured over time either in the presence of or without mevastatin treatment (asterisks denote differences between treatment groups, *denotes *P* < 0.05, **denotes *P* < 0.01). The graphs depict the means ± SEM of at least four separate experiments performed in duplicate.

Cultured GC and TC, treated with Mevastatin (final concentration of 5 μM) had less *LHR* mRNA than untreated cells (significant effect of treatment, *P* < 0.001; Fig. 7a and b) throughout the time course of the study. However, under the same treatment, the amount of aromatase (*CYP19*) mRNA (Fig. 7c) in GC was similar to that of the non-treated cells throughout the time of culture.

## 4 Discussion

The present study sought to further elucidate the mechanisms underlying the regulation of LHR in the somatic compartments of growing follicles. Perceived differences in LH sensitivity and LHR production in TC and GC suggest that local regulation is intricate and dynamic. Despite keen research interests, a full understanding of LHR regulation remains to be described. To further address this shortfall of understanding, profiles of relative amounts of *LHR* mRNA splice variants were assessed in ovarian somatic cells of cattle during gonadotrophin-induced differentiation *in vivo* and *in vitro*. Results from the present study of global LHR mRNA profiling at different stages of follicle development in cattle confirm and extend previous findings showing that TC from follicles at all antral stages and GC from large antral follicles have abundant amounts of *LHR* mRNA (Abdennebi et al., 2002, Bao et al., 1997, Peng et al., 1991) or protein (Carson et al., 1979, Webb and England, 1982). The presence of *LHR* mRNA in GC from small and medium sized follicles has also been previously reported using PCR based approaches (Robert et al., 2003). Although the present results support these previous observations, it is not certain whether the minimal amounts of these transcripts in GC of follicles <6 mm have any biological significance.

A novel observation of the present study with cattle was that in large antral follicles *LHR* mRNA in TC appears to decrease coincident with an increase in the GC of follicles in the same size group. However, as the follicles were selected without knowledge of physiological status this outcome must be viewed with caution. Additionally, the decrease in *LHR* mRNA in TC was significant only in 3′ VDS and not 5′ VDS variants and in another study (Simoes et al., 2012) TC of follicles (7 mm to >10 mm) from Nelore cattle, amounts of *LHR* mRNA did not change with increased follicle size. In contrast, the pattern of *LHR* mRNA in GC in the present study is supported by Simoes et al. (2012) where in Nelore cattle amounts of *LHR* mRNA was greater in large (>10 mm) follicles compared with smaller follicles 10 mm or less in size. These findings in GC were not unexpected because it is well established that large antral follicles are highly steroidogenic and LH-mediated androgens in the TC are an important substrate for aromatisation to oestrogens by the GC of developing follicles (McNatty et al., 1984). In mice, LHR-mediated progesterone production in the GC of large pre-ovulatory follicles is a pre-requisite for ovulation (Lydon et al., 1995, Karlsson et al., 2010) and that progesterone and not oestrogen may be preferentially produced by GC of large pre-ovulatory follicles of cattle (Conley et al., 1995).

A further major finding with the present research was that LHR mRNA in undifferentiated GC and TC from small antral follicles was up regulated with increasing time in culture in the presence of optimal doses of gonadotrophins, insulin and IGF-1 previously shown to induce steroid (Campbell et al., 1996, Campbell et al., 1998, Campbell and Baird, 2001, Gutierrez et al., 1997) and inhibin A (Campbell and Baird, 2001) production. Further, these induction profiles parallel those previously observed for other markers of GC differentiation (*FSH-R*, *CYP19*) during culture with an initial decrease, associated with dispersion and plating of the cells during culture, followed by an induction phase that preceded steroidogenic activity (Marsters et al., 2003). In poly-ovulating species (Erickson et al., 1982, LaBarbera and Ryan, 1981, Tilly et al., 1992), FSH induces LHR in cultured GC and in ruminants FSH-priming is required to induce follicle development to the stage that LHR develop in the GC of large antral follicles *in vivo* (Campbell et al., 2003, Webb et al., 1999). However, there are few reports of FSH-induced up-regulation of *LHR* mRNA in cultured GC from mono-ovulating ruminants and this finding reflects the problem of spontaneous luteinisation of cultured GC and TC that has proven to be difficult to overcome in ruminants (Campbell et al., 1996, Campbell et al., 1998, Gutierrez et al., 1997). However, this may be an important finding as it adds further support to the contention that, in this mono-ovulating species, LH-responsiveness may be important during the terminal stages of antral follicle development. Indeed, LH responsiveness has been strongly linked to a corresponding increase in ovulation capacity in Nelore cattle (Simoes et al., 2012).

In contrast to the positive effect of FSH on the induction of LHR in GC, it was the absence of LH in TC that appeared to mediate a marked increase in *LHR* mRNA after 96 h of culture. This is consistent with a negative feedback mechanism, for up-regulating LHR, being active in these cells to counteract a loss of LH-dependant events. It is tempting to speculate that there could also be a link with the steroidogenic pathway as decreasing amounts of *LHR* mRNA have been reported to follow parallel decreases in luteal and circulating concentrations of progesterone (Forni et al., 2003). The fact that removal of FSH from the GC media led to a >5-fold reduction in amounts of *LHR* mRNA suggests a direct regulatory relationship between FSH and *LHR* mRNA in GC and studies in rats indicate protein kinase B is an essential component of FSH-mediated GC differentiation (Zeleznik et al., 2003). Overall, the present results confirm that gonadotrophins are essential for sustaining *LHR* in follicular somatic cells of cattle and support the contention that this culture system stimulates the differentiation of ovarian somatic cells *in vitro* in a manner that parallels the process that occurs within ovarian follicle growth and maturation in response to gonadotrophic stimulation *in vivo*.

In addition to being able to precisely regulate culture conditions, a major advantage of the cell culture approach is the ability to examine temporal changes between gene expression and steroidogenic activity. In both TC and GC, *LHR* mRNA decreased when steroid production was maximal, again suggesting a local feedback mechanism regulating *LHR* mRNA. In addition, dissimilar *in vitro* profiles of *LHR* mRNA in GC and TC paralleled the *in vivo* findings in the present study. Amounts of *LHR* mRNA in TC reached a peak within 48 h of culture and remained stable for 96 h thereafter, whereas amounts in GC increased over a 96 h period to reach a transient peak. Thus it would appear that LHR production may be regulated in a pre-translational and tissue-specific way. One possibility proposed for specialised regulation is that *LHR* mRNA-binding proteins (LRBPs) may induce rapid LHR degradation (Kash and Menon, 1999). Kash and Menon identified an LRBP binding site adjacent to the transcription start site and demonstrated that LRBP/*LHR* mRNA complexes are more rapidly degraded than unbound *LHR* mRNA thereby limiting time for translation. LRBP has been identified as mevalonate kinase (MVK), an important enzyme in the *de novo* cholesterol synthesis pathway (Wang et al., 2007, Nair et al., 2008). This mechanism is active in human GC and it seems reasonable to surmise that this mechanism would prevent accurate measurement of amounts of mRNA by PCR methodology (Wang et al., 2007). The precise amount of *LHR* mRNA would not be able to be determined as the LRBP/*LHR* mRNA complexes would either block cDNA primer access or dramatically reduce the target pool due to rapid LHR degradation. In either event the outcome would be the apparent loss of mRNA. Though this present work requires further corroboration it does add support that the negligible amounts of *LHR* mRNA detected in GC of immature follicles may be due, in part, to the post-transcriptional influence of an LRBP mechanism. In the presence of mevastatin (a chemical used to block the *de novo* cholesterol synthesis pathway), *LHR* mRNA amounts in both cultured GC and TC of cattle, were minimal. This appeared to be an *LHR*-specific effect as cell growth continued apparently unaffected and expression of a marker gene, aromatase, was unaltered with this treatment. It is hypothesised that enriched cellular MVK, under these circumstances, may increase the rate of LRBP (MVK)/*LHR* mRNA complexing and thus inhibit LHR translation. This would imply that known *de novo* cholesterol availability differences in GC and TC are significant in the regulation of *LHR* translation.

Relative amounts of the 3′ VDS splice variants in TC of cattle follicles of all size groups showed that though amounts varied quite markedly across the different size follicle pools, differences between the variants in each pool were mostly non-significant. However, the fact that amounts of the ‘G’ variant were markedly less than the other variants in all follicle size groups but was similar to amounts of the other isoforms in the cultured TC does suggest a culture-specific effect. Furthermore, a similar effect seems apparent in the GC cultures where the amounts of the ‘G’ form variant were either non-existent or markedly less than the other forms in cells taken directly from different size follicles, but similar to amounts of the other isoforms in the cultured GC. It is evident that splice variants containing deletions in the 5′ VDS are substantially less in somatic cells throughout antrum follicle development than in those cells with this site intact. The exception to this is the previously reported (Nogueira et al., 2007) 5′ VDS variant which lacks Exon 3 in its entirety. This variant was detected in present study in TC from follicles ≥1 mm and GC from follicles >10 mm in amounts that infer biological relevance. In these cells, the intact 5′ VDS form was the major splicing event but in addition this minor 5′ VDS form that has the entire Exon 3 deleted, was also present in these cells. However, the patterns of these splice variants in cultured TC and GC when compared to the patterns *in vivo* add more support for the involvement of a regulatory mechanism which is culture-specific. In both cell-types cultured over time, isoforms with an intact 5′ VDS were greatly elevated over those lacking Exon 3 (around 10-fold) compared to in the *in vivo* cells (around two-fold). This suggests that Exon 3 may be an important component in the regulation of 3′ VDS variants. Moreover, because this region of the gene encodes the ligand specific section of the LHR ectodomain (Remy et al., 1996), it is reasonable to hypothesise that this function would be impaired in putative protein products of transcripts with deletions in the 5′ VDS.

Taken together, these outcomes do nothing to dispel the idea, expounded on previously (Bacich et al., 1999), that variability of putative variant proteins would involve other post-transcriptional regulatory mechanisms. The present study supports the contention that ‘A’, ‘F’, and ‘B’, 5′variants may be important because of the large amounts in TC from all follicles >1 mm and in GC from follicles >10 mm but at a markedly lesser amount in GC from follicles between 6 and 10 mm. The ‘G’ form of mRNA was in lesser amounts but may have a role in TC. The ‘A’ form variants encode LHR ectodomains with both membrane spanning and nuclear signalling regions and include the full-length fully functional receptor form. It is tempting to suggest functional roles for members that were present in larger amounts of these variant families. The *LHR* ‘F’ mRNA variants have all of Exon 10 deleted. Putative protein products may have a reduced hormone-binding ability as Exon 10 contains three conserved putative N-linked glycosylation sites (Zhang et al., 1995). Continued exposure to LH can lead to a desensitisation of the LH-response (West and Cooke, 1992). It is, therefore, suggested that a possible mechanism for this control is variation of receptor affinity that may, in part, be provided by the precise regulation of the ratio of ‘A to F’ forms.

From these data, it is hypothesised that changes in LH-responsiveness of follicular somatic cells is due to multiple regulatory mechanisms. Data from the presents study confirm that LHR mRNA is present in TC during the stages of follicular development examined but amounts are only greater than background in GC from large follicles. However, a limitation of the present study was that, though only follicles appearing to be morphologically normal were selected, the precise physiological status of the follicles was not known at the time of their collection. The present cell culture studies also indicate that amounts of *LHR* mRNA differ in GC and TC during *in vitro* gonadotrophin-induced cellular differentiation and that *LHR* in GC and TC is differentially regulated by gonadotrophins, adding further support to cell type-specific regulation of *LHR* mRNA. A number of *LHR* mRNA isoforms in follicular somatic cells of cattle were also detected in the present study, which vary due to differential splicing of the 5′ and 3′ VDS. These include members of the ‘A’, ‘F’, ‘B’, and ‘G’ 3′ VDS variant families along with a novel 5′ VDS variant (Nogueira et al., 2007). It is concluded that amounts of somatic cell *LHR* mRNA is controlled by intricate local mechanisms and that the serum-free somatic cell culture systems used in the present study are ideally suited to elucidate these control mechanisms and to determine the physiological role of *LHR* mRNA splice variants in mono-ovulating species.

## Conflict of interest

The authors declare that there is no conflict of interest that would prejudice the impartiality of this scientific work.

## Figures and Tables

**Table 1:** *LHR* sequence-specific primers used in conventional qPCR amplification and identification of variably deleted sequence. The primer denotations and sequences are given.

Primer	Sequence	Orientation
LHR.ex1.F	5′-GCCTCAGCCGACTATCACTC-3′	Forward
LHR.ex7.F	5′-AATGGGACAACGCTGATTTC-3′	Forward
LHR.ex8.R	5′-TCGTTGTGCATCTTCTCCAG-3′	Reverse
LHR.ex9.R	5′-GGGGTAAGTCAGTGTGGCAT-3′	Reverse
LHR.ex11.R	5′-CATGGTTGTAATACTGGCCT-3′	Reverse
18S rRNA.F	5′-TTGACTCAACACGGGAAACT-3′	Forward
18S rRNA.R	5′-AGAAAGAGCTATCAATCTGTCAATCCT-3′	Reverse
